# Sensory neuronopathy in a patient with neurofibromatosis type 1: A case report

**DOI:** 10.1097/MD.0000000000031718

**Published:** 2022-11-11

**Authors:** You-Ri Kang, Seong-Min Hong, Jong-Hee Choi, Seung-Jin Lee, Jae-Myung Kim, Kyung Wook Kang, Tai-Seung Nam

**Affiliations:** a Department of Neurology, Chonnam National University Hospital, Gwangju, Korea; b Department of Radiology, Chonnam National University Bitgoeul Hospital, Gwangju, Korea; c Department of Neurology, Chonnam National University Medical School, Gwangju, Korea.

**Keywords:** ataxia, meningocele, neuroendocrine tumors, neurofibromatosis 1, spinal ganglia

## Abstract

Neurofibromatosis type 1 (NF-1) can manifest with various neurological symptoms. However, sensory ataxia has not been reported.

**Patient concerns:**

A 44-year-old man with NF-1 presented with several weeks of unsteady gait. He was diagnosed with gastric neuroendocrine tumor with multiple hepatic metastases 6 years ago and received palliative chemotherapy. Neurological examination revealed ataxia veering to the right side with no motor weakness.

**Diagnoses:**

Clinical manifestations and electrodiagnostic studies suggested the dysfunction of the thoracic dorsal column (DC). Initial magnetic resonance imaging showed a lateral thoracic meningocele (LTM) located in the right paravertebral area at the T3-T4 vertebral level, but the spinal cord was unremarkable. Gait disturbance worsened after 9 months, and follow-up magnetic resonance imaging showed high signal intensity involving the right DC at the level adjacent to the LTM and spinal cord atrophy distal to the DC lesion. Tests for well-characterized paraneoplastic antibodies were negative. Ultimately, the patient was assumed to have sensory neuronopathy due to compressive damage to the dorsal root ganglia within the intervertebral foramina by LTM.

**Interventions:**

Empirical treatment with vitamin B12 supplementation and corticosteroids failed to improve his condition. The patient underwent decompressive laminectomy and excision of the meningocele with dura repair.

**Outcomes:**

The patient temporarily improved to walk with assistance postoperatively. However, he developed dyspnea and hypotension 5 weeks later. Carcinoid heart disease confined the patient to the bed. The patient died of pneumonia 3 months after the operation.

**Lessons:**

This case with NF-1 shows asymmetric sensory ataxia of subacute progression. LTM may contribute to the development of sensory neuronopathy by damaging sensory neurons of the dorsal root ganglia. The comorbidities of the patient, including gastric neuroendocrine tumor and LTM, made it challenging to investigate the pathomechanism.

## 1. Introduction

Neurofibromatosis type 1 (NF-1) is an autosomal dominant multi-systemic disorder characterized by cutaneous features such as multiple café-au-lait spots, freckles, and neurofibromas.^[1]^ It is associated with NF1 tumor suppressor gene mutations, and the affected individuals are prone to developing benign or malignant neoplasm.^[2]^ Meanwhile, NF-1 patients can present with various neurological manifestations,^[3]^ and some of them could be caused by the involvement of the spinal nerves or adjacent structures by paravertebral neurofibroma, kyphoscoliosis, or lateral thoracic meningocele (LTM).^[4–6]^ However, to the best of our knowledge, sensory ataxia has not yet been reported in patients with NF-1. We recently experienced an NF-1 patient with LTM and gastric neuroendocrine tumor (NET) who presented with sensory ataxia of subacute progression. The patient was finally diagnosed with sensory neuronopathy (SNN), but the scarcity of previous reports and the patient’s comorbidities made it challenging to investigate the etiology.

## 2. Case presentation

A 44-year-old man with NF-1 presented with a several weeks’ history of unsteady gait. Since adolescence, he had multiple café-au-lait spots and neurofibromas, and NF-1 was confirmed by identifying a pathogenic heterozygous splicing mutation (c.2410-16A > G, 2409ins15). The patient received 14 cycles of palliative combination chemotherapy with cisplatin and etoposide for about 4 years due to gastric NET with multiple hepatic metastases since he was 38. During chemotherapy, he complained of paresthesia in the fingertips and tiptoes on both sides, which was diagnosed as chemotherapy-induced peripheral neuropathy. His malignancy had been stable for over 2 years without further chemotherapy on regular checkups, but he continued receiving a monthly somatostatin analogue injection due to chronic diarrhea.

**Figure 1.**
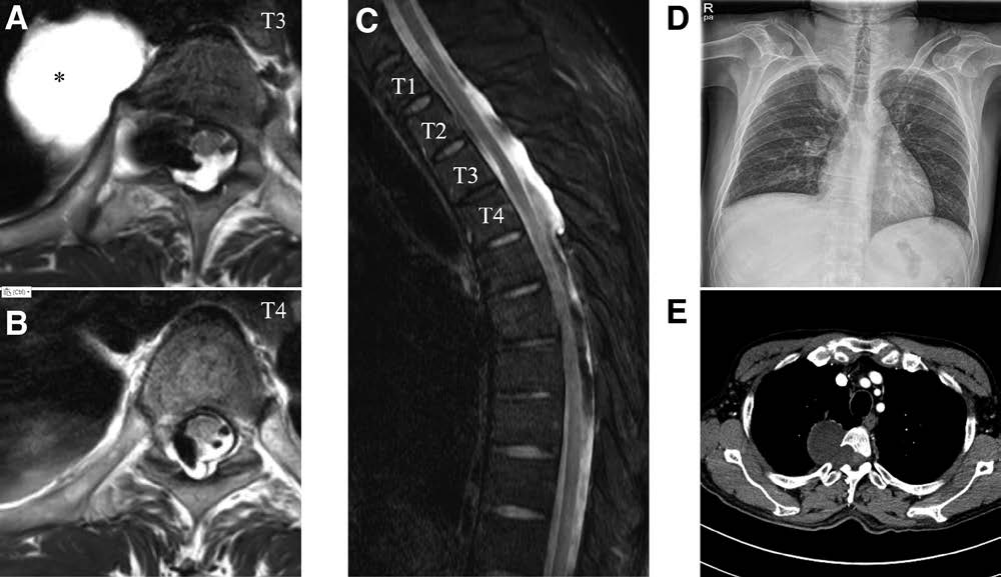
Initial radiologic findings of the patient. Axial and sagittal T2-weighted MR images show a 4.5 × 5.0 × 4.8 cm sized mass with high signal intensity (black asterisk) in the right paravertebral area at the T3-T4 vertebral level, and the normal-appearing spinal cord (A–C). Chest X-ray shows an oval high-density lesion in the upper right lung field (D). Chest computed tomography shows a dumbbell-shaped low-density mass lesion with the bony remodeling of the T3-T4 vertebrae and widened intervertebral foramen (E). MR = magnetic resonance.

Neurological examination revealed ataxia veering to the right side while walking with no motor weakness. Vibration sensation was significantly impaired in the right leg, but pain/temperature sensation was relatively spared in the 4 limbs. Leg somatosensory evoked potential (SEP) showed increased latencies of peripheral and central conduction time on the right side, while the result of arm SEP was normal. Nerve conduction study showed symmetric distal sensory axonopathies in the 4 limbs, but it showed no interval change compared with a previous study performed when he was diagnosed with chemotherapy-induced peripheral neuropathy. Dorsal column (DC) myelopathy was suspected, but magnetic resonance imaging (MRI) of the spine showed no signal abnormality in the spinal cord. Instead, a non-enhancing cystic mass was observed in the paraspinal area (Fig. 1A–C). Chest radiograph and computed tomography (CT) revealed a dumbbell-shaped LTM protruding through an enlarged T3-T4 intervertebral foramen on the right side (Fig. 1D and E). An extensive serological workup, including serum vitamins B6 and B12, homocysteine, methylmalonic acid, autoantibodies (anti-antinuclear antibody, ganglioside antibodies, anti-intrinsic factor antibody, anti-parietal cell antibody, anti-aquaporin-4 antibody, and anti-SS-A/B antibodies), and viral markers (varicellar zoster, herpes simplex virus, human immunodeficiency virus, human T-lymphotropic virus type 1, and syphilis), produced unremarkable results. Onconeural antibodies, including anti-Hu, anti-Ri, anti-Yo, anti-amphiphysin, anti-CV2, anti-PNMA2, anti-recoverin, anti-SOX1, anti-titin, and anti-glutamic acid decarboxylase, were all negative. Cerebrospinal fluid (CSF) analysis was unremarkable, and no malignant cells were identified. The patient was empirically treated with vitamin B12 and corticosteroids for several months but failed to improve.

**Figure 2.**
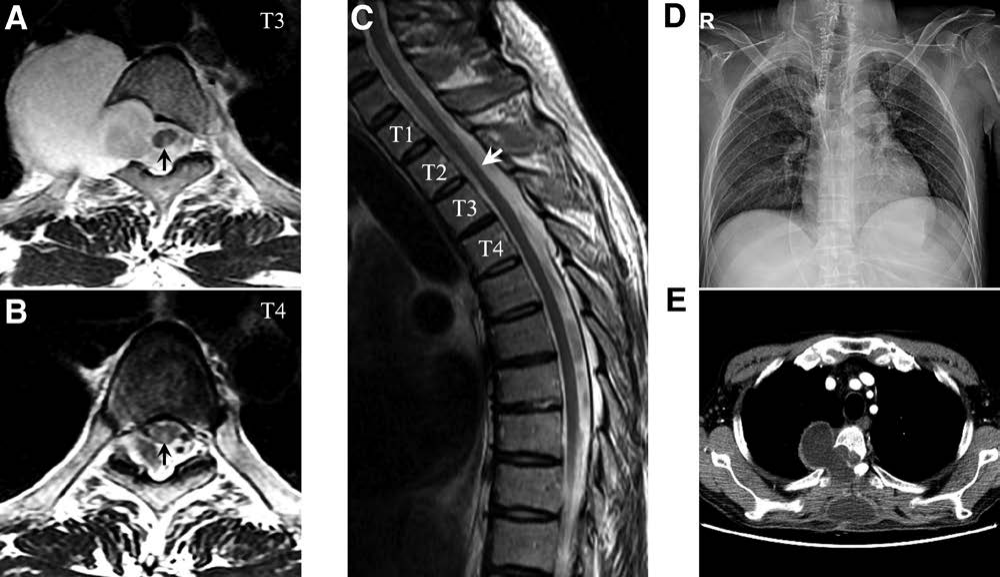
Follow-up radiologic finding 9 mo after the onset. Axial T2-weighted MRI shows focal hyperintensity (black arrows) involving the dorsal column of the spinal cord at the vertebral level of T3-4 (A and B). Midsagittal T2-weighted MRI shows spinal cord atrophy (white arrow) at the vertebral levels T2-T3 (C). Chest X-ray after decompression surgery shows no more oval opacity (D). Chest CT shows interconnected two peripherally-enhancing fluid collections in the paravertebral area and upper back (E). CT = computed tomography, MRI = magnetic resonance imaging.

Nine months after symptom onset, the patient complained of paresthesia in the right leg, and his ataxic gait worsened. Thoracic MRI showed T2 high signal intensity involving the right DC without contrast enhancement, mainly at the T3-T4 vertebral levels (Fig. 2A and B), and segmental spinal cord atrophy distal to the DC lesion (Fig. 2C). The size of the LTM did not expand, and the malignancy did not progress on the CT of the chest and abdomen. A newly seen DC lesion was presumed to be a subsequent change following damage of the dorsal root ganglia (DRG), that is, SNN. Repeated testing for paraneoplastic antibodies yielded negative results. Based on these investigations, chronic mechanical pressure was assumed to cause DRG injury, considering the spatial relationship between the DC lesion and LTM. Thus, the patient underwent laminectomy with dural repair after meningocele isolation (Fig. 2D). He partially improved his ability to walk several meters with assistance and was transferred to the rehabilitation center. However, 5 weeks later, the patient developed dyspnea and refractory hypotension due to right heart failure, which turned out to be a carcinoid heart disease on the echocardiogram. Follow-up CT showed 2 communicating fluid collections with peripheral rim enhancement in the right paravertebral area and the upper back (Fig. 2E). His general condition worsened rapidly, and he was confined to the bed. Despite the best medical therapy, the patient died of pneumonia 3 months after the operation.

## 3. Discussion

This report discusses an NF-1 patient with LTM who presented with gait disturbance. In the previous literature, a case of a patient with NF-1 who had progressive paraparesis due to a giant LTM compressing the spinal cord has been reported.^[5]^ However, sensory ataxia has never been reported in patients with NF-1, especially in association with LTM. In the current case, the patient showed asymmetric sensory ataxia of subacute onset, abnormal leg SEP, and signal change within the DC on MRI. Based on these findings, the patient was diagnosed with *probable* SNN according to the criteria proposed by Camdessanché et al.^[7]^

SNN is a rare neurological disorder characterized by the primary degeneration of sensory neurons of the DRG and subsequent impairment of its central and peripheral projections.^[8]^ Regarding the etiology, half of the cases of SNN are idiopathic, 20% are paraneoplastic, and the rest are linked to Sjögren’s syndrome, pyridoxine toxicity, platinum-based chemo-agent toxicity, and viral infection.^[8–11]^ Our patient had factors related to SNN, such as cisplatin and malignancy, but it was not evident that they were the leading cause. There seemed to be little temporal correlation between cisplatin chemotherapy and SNN in that ataxia developed 2 years after the completion of chemotherapy, even considering the coasting phenomenon.^[12]^ Furthermore, SNN is not commonly associated with NETs,^[13]^ and only 1 case with anti-Hu antibody in a patient with gastric NET has been reported to date.^[14]^ In our case, sensory ataxia developed as much as 6 years after the cancer diagnosis, and was confined to the right leg without spreading to the upper limbs over time. CSF examination results were also unremarkable, while CSF inflammation is known to be common in paraneoplastic neurological syndromes.^[15]^ In addition, no well-characterized onconeural antibodies were detected.

Intriguingly, the focal lesion in the DC on MRI was located ipsilaterally adjacent to the LTM, which may provide a clue for inferring the pathomechanism of SNN in our case. Although LTM is often asymptomatic, it can cause symptoms such as dyspnea, radicular pain, or motor weakness due to local compression of the lung, peripheral nerves, or spinal cord, depending on its size and location.^[16]^ In this context, we assumed that mechanical compression by the LTM might have damaged the cell bodies of sensory neurons of the DRG or their central processes, and the segmental spinal cord atrophy could be explained as centripetal Wallerian degeneration following damage to the DRG.^[17]^ Although the LTM is not very huge, DRG has been reported to be susceptible to compressive damage.^[18]^ Moreover, even though the neurotoxic effect of cisplatin on the DRG was not enough to cause overt symptoms after chemotherapy, prior cisplatin use might have made the sensory neurons of the DRG more vulnerable to additional injury due to direct compression or local ischemia by a space-occupying lesion, such as LTM. This study had several limitations. A biopsy of the DRG was not performed, and the long-term surgical outcome could not be assessed adequately because the patient died early after the operation.

In summary, this case showed that LTM might cause SNN due to local compression of the DRG in patients with NF-1. Comorbidities have made it difficult to determine the etiology of sensory ataxia. Further studies are needed on neurological complications in NF-1 patients.

## Author contributions

**Conceptualization:** Tai-Seung Nam.

**Data curation:** You-Ri Kang, Seong-Min Hong, Jong-Hee Choi, Jae-Myung Kim.

**Formal analysis:** You-Ri Kang, Kyung Wook Kang, Seung-Jin Lee.

**Investigation:** You-Ri Kang, Tai-Seung Nam.

**Writing – original draft:** You-Ri Kang, Tai-Seung Nam.
